# The toxic natural product tutin causes epileptic seizures in mice by activating calcineurin

**DOI:** 10.1038/s41392-023-01312-y

**Published:** 2023-03-10

**Authors:** Qing-Tong Han, Wan-Qi Yang, Caixia Zang, Linchao Zhou, Chong-Jing Zhang, Xiuqi Bao, Jie Cai, Fangfei Li, Qinyan Shi, Xiao-Liang Wang, Jing Qu, Dan Zhang, Shi-Shan Yu

**Affiliations:** State Key Laboratory of Bioactive Substances and Functions of Natural Medicines, Institute of Materia Medica, Peking Union Medical College and Chinese Academy of Medical Sciences, Beijing, 100050 China

**Keywords:** Target identification, Diseases of the nervous system

## Abstract

Tutin, an established toxic natural product that causes epilepsy in rodents, is often used as a tool to develop animal model of acute epileptic seizures. However, the molecular target and toxic mechanism of tutin were unclear. In this study, for the first time, we conducted experiments to clarify the targets in tutin-induced epilepsy using thermal proteome profiling. Our studies showed that calcineurin (CN) was a target of tutin, and that tutin activated CN, leading to seizures. Binding site studies further established that tutin bound within the active site of CN catalytic subunit. CN inhibitor and calcineurin A (CNA) knockdown experiments in vivo proved that tutin induced epilepsy by activating CN, and produced obvious nerve damage. Together, these findings revealed that tutin caused epileptic seizures by activating CN. Moreover, further mechanism studies found that *N*-methyl-*D*-aspartate (NMDA) receptors, gamma-aminobutyric acid (GABA) receptors and voltage- and Ca^2+^- activated K^+^ (BK) channels might be involved in related signaling pathways. Our study fully explains the convulsive mechanism of tutin, which provides new ideas for epilepsy treatment and drug development.

## Introduction

Epilepsy, a common paroxysmal chronic nervous system disease, is accompanied by various features, such as cognitive dysfunction and neuronal necrosis.^1,2^ It is thought that epilepsy is induced by a dysfunctional imbalance between excitatory and inhibitory neurotransmitters. The etiology of epilepsy is complex, and its pathogenesis has not been completely clarified. Increasing evidence suggests that multiple interacting factors influence seizure intiation.^3^ Previous studies have shown that pyramidal neuronal loss and degeneration are common features in various regions of the hippocampus in animal models of epilepsy.^4,5^ Anti-epileptic drug development has been hindered since the etiology of epilepsy has not yet fully elucidated. Therefore, uncovering the pathogenesis and molecular potential targets of epilepsy is an urgent need with great significance for the treatment of epilepsy.

Natural products are crucial sources of the discovery of new drugs, and increasing numbers of studies have suggested that authenticating underlying targets of natural products is a vital approach to fully elucidate the mechanism of their actions. Recently, more and more target proteins of natural products have been screened using chemical proteomic approaches.^6,7^ For example, researchers have conducted in-depth research on the mechanism of metformin through chemical biology methods, and found that metformin plays its role by targeting adenosine monophosphate-activated protein kinase (AMPK) signaling pathway via presenilin enhancer 2 (PEN2).^8^ Molecular targets found in epilepsy caused by toxic natural products may also provide new ideas and strategies for the treatment of the disease. Currently, the natural products that can induce epilepsy include pilocarpine and Coriaria lactone (CL). CL, extracted from *Coriariaceae*, has been used to treat schizophrenia with seizures. Unsurprisingly, toxicity studies indicated that CL-treated animals exhibited symptoms of seizures, muscle spasticity and respiratory paralysis.^9,10^ Tutin, an established toxic compound, was first isolated and identified as a convulsive poison in the leaves and seeds of the New Zealand species of *Coriaria*.^11,12^ Tutin is the main epileptogenic component of CL, and perfusion of it induced or increased discharge from nucleus tractus solitarius neurons in vitro.^13^ Tutin overstimulates the nervous system, leading to hyperactivity and seizures.^14^ Previous studies on the tutin mechanism of action found that tutin-induced epilepsy may be related to inhibition of gamma-aminobutyric acid (GABA) receptor and glycine, but the specific mechanism has not been clarified.^9,15,16^ To discover the molecular targets of epilepsy and prevent the occurrence of the disease, the toxicity mechanism of tutin urgently needs to be elucidated, which will contribute to the development of antiepileptic drugs.

In this study, a series of experiments were designed to identify the mechanism of tutin action. It was found that tutin induced seizures through targeting the catalytic subunit A (CNA) of calcineurin (CN) via thermal proteome profiling-temperature range (TPP-TR) approach and hydrogen deuterium exchange mass spectrometry (HDX-MS) methods. These findings further validated that CN might be a possible potential target of tutin by employing a CN inhibitor (FK506) and CNA knockdown in vivo.

## Results

### Epilepsy induced by tutin is antagonized by diazepam and MK-801 in mice

**Fig. 1:**
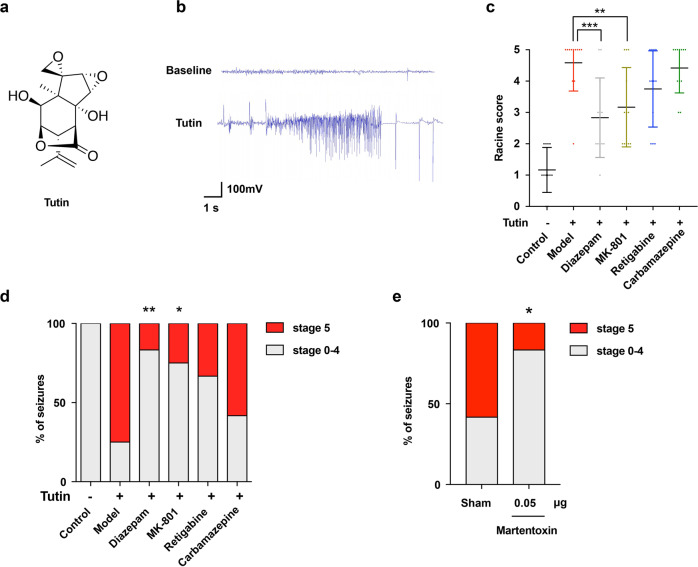
Epilepsy caused by tutin is antagonized by diazepam and MK-801 in mice. **a** Chemical structure of tutin. **b** EEG (1-min) tracings during the pre-injection baseline and tutin injection. **c** Effects of antiepileptic drugs on maximum Racine score within 2 h after tutin injection. Results were represented as mean ± SD with *n* = 12, **P < 0.01, ***P < 0.001 vs. model group. **d** Effects of antiepileptic drugs on the intensity of the seizure attack in mice (*n* = 12). *P < 0.05, **P < 0.01 vs. model group. **e** Effect of martentoxin on the intensity of the seizure attack in mice (*P < 0.05 vs. sham group, *n* = 12)

Tutin is a well-known epileptogenic agent whose chemical structure is shown in Fig. 1a. We evaluated its half convulsive dose (CD_50_) and half lethal dose (LD_50_), and verified that tutin can cause seizures in mice (Supplementary Fig. S1a, b), Moreover, the electroencephalography (EEG) signal of tutin-treated mice was detected in the present study. Consistent with previous studies,^13^ tutin-treated mice exhibited typical epileptic EEG (Fig. 1b). Microdialysis with LC-MS/MS experiment was used to investigate changes of seizure-related neurotransmitters. This result indicated that tutin induced increased glutamate (Glu)/GABA ratio in the intercellular space (Supplementary Fig. S2). Meanwhile, Glu/GABA ratio was higher in epileptic mice, supporting that brain hyperexcitability is a vital feature of epilepsy induced by tutin. To explore the underlying mechanism that causes epilepsy, we selected antiepileptic drugs with different mechanisms, including diazepam, MK-801, retigabine and carbamazepine, and observed their therapeutic effects on epilepsy induced by tutin (2 mg/kg, intraperitoneally injected [i.p.]). It is known that Diazepam alleviates seizures by enhancing GABA-mediated inhibitory neurotransmission, and MK-801 antagonizes *N*-methyl-*D*-aspartate (NMDA) receptor.^17,18^ Retigabine exerts anti-epileptic effects through inhibition of neuronal excitation via voltage-gated KCNQ2-5 potassium channel activation,^19^ and carbamazepine is a voltage-dependent sodium channel blocker.^20–22^ The results showed that pretreatment with diazepam or MK-801 significantly decreased the maximal seizure score in 2 h (Fig. 1c, d), indicating that diazepam and MK-801 alleviated the epileptic behavior of mice induced by tutin. However, the effects of retigabine and carbamazepine on seizures were not obvious. In addition, martentoxin, a voltage- and Ca^2+^- activated K^+^ (BK) channel blocker,^23,24^ alleviated tutin-induced severe seizures in mice (Fig. 1e). Therefore, we speculated that the mechanisms of tutin-induced epilepsy in mice might be related to NMDA receptors, GABA receptors and BK channels.

### Identification of tutin-targeting proteins in primary cultured hippocampal neurons of rats is conducted via TPP-TR approach

**Fig. 2:**
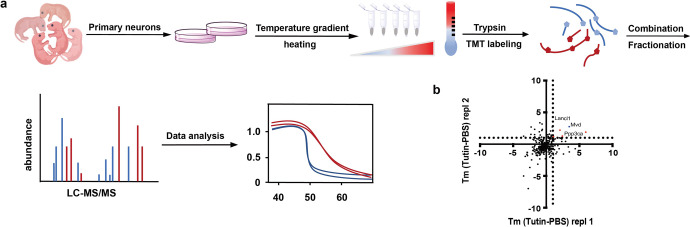
Target identification of tutin in primary hippocampal neurons is conducted using TPP-TR. **a** Schematic procedure of TPP-TR. **b** Scatterplot of Tm shifts of tutin vs. PBS treatment. The TPP R package was used for calculating the thermal response curve fitting and melting point

Activity-based protein profiling (ABPP) is regarded as an important approach to identify the potential targets of natural products.^25–27^ We firstly performed structural modification of tutin, such as hydroxyl modification into probe with terminal alkyne group (Supplementary Figs. S3-S5), ethylene oxide opening, and lactone ring opening.^28^ However, the ability of this modified tutin to induce epilepsy was decreased significantly, which makes this method unsuitable for the targets study of tutin targets (Supplementary Tab. S1). Hence, TPP as an alternative modification-free strategy, was used to study the target of tutin in primary hippocampal neurons.^29,30^ The primary hippocampal neurons were incubated in situ with 5 μM of tutin or PBS for 3 h, and then exposed to differnent temperatures for 3 min. The isolated proteins were labeled by tandem mass tag (TMT),^31^ which were exposed to UPLC fractionation and assayed by LC-MS/MS^32^ (Fig. 2a). Tm shifts were calculated on the basis of two replicates of tutin compared to PBS treatment and visualized after filtering (Fig. 2b). The minimum temperature shift of 1 °C was identified for 36 proteins in both the tutin- and PBS-treated replicates. Only diphosphomevalonate decarboxylase (Mvd) met additional significance thresholds (see the blue dot and melting curve in Supplementary Fig. S6). In addition to the significance thresholds, four additional proteins satisfied all other conditions (Supplementary Tab. S2): serine/threonine-protein phosphatase 2B catalytic subunit alpha isoform (ppp3ca), glutathione S-transferase LANCL1 (Lancl1), aldehyde dehydrogenase family 3 member B1 (Aldh3b1) and hydroxysteroid dehydrogenase-like protein 2 (Hsdl2). As determined with an extensive literature review, Mvd, Lancl1, Aldh3b1 and Hsdl2 have not been found to be closely related to epilepsy. CNA has been regarded as a potential molecule of epilepsy, and reported to participate jointly in epilepsy and regulate the activities of GABA and NMDA receptors.^33,34^ Moreover, we have found that GABA and NMDA receptors may be involved in epilepsy induced by tutin since antiepileptic drugs (diazepam and MK-801) showed significant antagonism effects (Fig. 1c, d). Collectively, all the data give us a hint that CNA may be a possible target of tutin.

### Tutin binds to CN

**Fig. 3:**
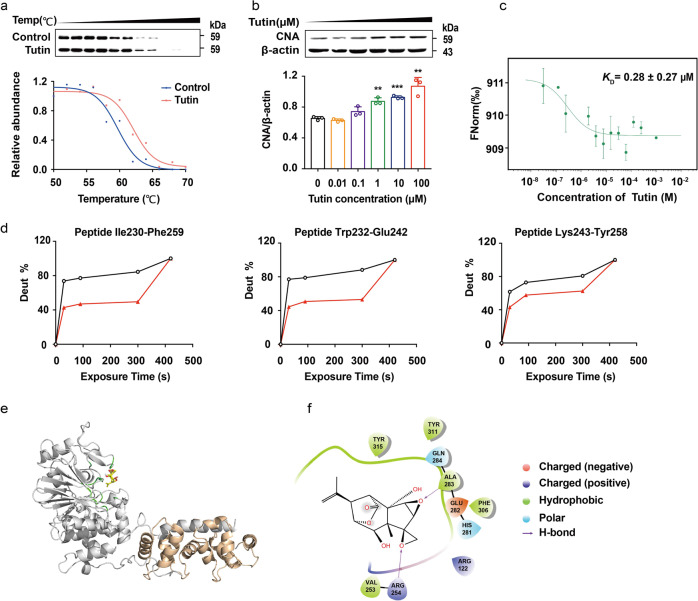
Tutin binds to CN. **a** Tutin increases the CNA thermal stability in living cells by temperature-dependent CETSA (*n* = 3). **b** Tutin increases the CNA thermal stability in living cells by concentration-dependent CETSA. Results were expressed as mean ± SD with *n* = 3. **c** The MST dose-response curve shows the interaction between tutin and CN. Results were expressed as mean ± SD with *n* = 3. **d** Deuterium uptake of peptides targeted by tutin. HDX graphs of CN alone (black curves) and bound both to tutin (red curves). **e** Structural overview of a predicted CN-tutin complex model (CNA: gray, CNB: orange, tutin: yellow, residues: green). Zoom-in view of the predicted CN-tutin interface. **f** Interface residues (Arg 254 and Ala 283) in CN are shown and labeled by the names and positions of residues

CN is composed of catalytic subunits (atalytic domain, CNB binding domain, calmodulin-binding domain and autoinhibitory domain) and regulatory subunits.^35,36^ Mutants lacking the latter calmodulin-binding domain and autoinhibitory domain exhibit constitutively active phosphatase activity independent of Ca^2+^/calmodulin.^37^ In this study, different methods were applied to investigate whether tutin interacts with CN. Firstly, Western blot-based cellular thermal shift assay was applied to analyze the protein-tutin interactions in cells. Both temperature- and dose-dependent cellular thermal shift assay (CETSA) data revealed that tutin influcences the CNA thermal stability (Fig. 3a, b), but not the β-actin (Supplementary Fig. S7), demonstrating that there was an interaction between CN and tutin. The isothermal titration calorimetry (ITC) analysis showed insufficient heat absorption or release, indicating that the binding force of tutin and CN was low and that cocrystallization and cryo-scanning electron microscopy (cryo-SEM) were not suitable for the detection of CN-tutin interactions. Therefore, microscale thermophoresis (MST) experiments were further applied to investigate the interaction between tutin and CN, and the data demonstrated that tutin directly bound to CN with a dissociation constant (*K*_D_) of 0.28 ± 0.27 μM (Fig. 3c). To explore their possible binding sites of CN and tutin, we compared the profiles of CN alone and CN with tutin through HDX-MS.^38^ Interestingly, the HDX-MS results suggested that CN bound tutin. All peptide deuterium uptake profiles were assayed, and seven peptides were identified using LC-MS/MS. Treatment with tutin altered the rate of H/D exchange of seven peptides (peptides 12-46, 62-72, 81-86, 81-95, 230-259, 232-242, and 243-258) (Fig. 3d, Supplementary Tab. S3). Among these seven peptides, three obvious changed peptides (230-259, 232-242, 243-258) were located in the active site of the catalytic CN subunit, indicating that tutin bound to the active site of the catalytic subunit of CN.

Considering the results of the HDX-MS, we performed active-pocket molecular docking with Schrodinger’s soft. To further predict how tutin interacts with CN, we modeled tutin binding to CN crystal structure (PDB ID: 6NUU). The results showed that tutin exhibited excellent docking to CN. Tutin may anchor in a CN binding site via the following interactions. The hydrogen bonds formed between the ethylene oxide moiety in tutin and Arg 254 or Ala 283 in CN (Fig. 3e, f). To further confirm this conclusion, two mutations (CN-R254K and CN-A283V) have been created, and MST experiments were carried out. The MST data revealed that the binding force was significantly decreased after the mutations. The *K*_D_ of CN-R254K and tutin was 7.64 ± 4.48 μM and the *K*_D_ of CN-A283V and tutin was 70.8 ± 45.6 μM respectively (Supplementary Fig. S8), both are significantly higher than the *K*_D_ of wild type CN and tutin (0.28 ± 0.27 μM, showed in Fig. 3c). This result further confirms that tutin binds to Arg254 and Ala283 in CN.

### Tutin activates CN in vitro and in vivo experiments

**Fig. 4:**
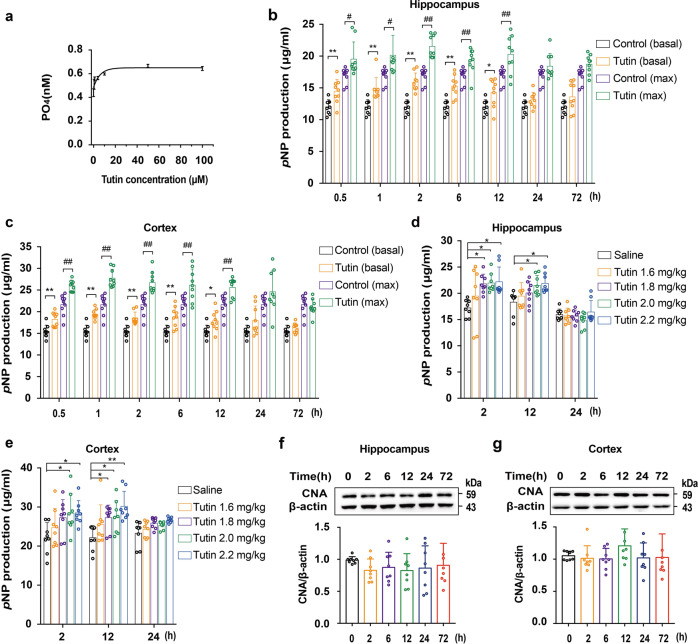
Tutin activates CN activity in vitro and in vivo. **a** Tutin activates CN in vitro, and results were presented as mean ± SD with *n* = 3. **b**, **c** Changes in CN activity after tutin-induced epilepsy. Basal and max CN activity of hippocampus or cortex were assayed (0.5, 1, 2, 6, 12, 24, 72 h). (*P < 0.05, **P < 0.01 vs. control group (basal). ^#^*P* < 0.05, ^##^*P* < 0.01 vs. control group (max.)). Results were represented as mean ± SD with *n* = 9. **d**, **e** Changes in CN activity of hippocampus or cortex after tutin-induced epilepsy. Mice were injected with different doses (0, 1.6, 1.8, 2.0, 2.2 mg/kg) of tutin, CN activity of hippocampus or cortex was assayed (2, 12, 24 h) (*P < 0.05, **P < 0.01 vs. saline group). Results were expressed as mean ± SD with *n* = 8. **f**, **g** CNA expression in hippocampus or cortex by Western blot. Results were represented as mean ± SD with *n* = 8

We have confirmed that tutin binds with the catalytic domain of CN. However, whether tutin affects the enzyme activity of CN was unclear. Therefore, whether tutin influences CN activity was measured in vitro. The results showed that tutin significantly activated CN in a dose-dependent manner in vitro (Fig. 4a). Moreover, CN activities in the hippocampal and cortical regions of mice was measured (0.5, 1, 2, 6, 12, 24 and 72 h) after seizures induced by tutin. The data revealed that significant increases in both basal and maximal CN activities were observed after the seizures, peaking at 0.5-6 h, and decreased to basal levels in the hippocampus and cortex after 24 h (Fig. 4b, c). Besides, to better visualize the dose-dependent effect of tutin on CN activity, mice were injected with tutin (0, 1.6, 1.8, 2.0, 2.2 mg/kg). And then CN activities in the hippocampus and cortex were observed at 2 h, 12 h and 24 h after seizures. The results indicated that tutin increased CN activity in a dose-dependent manner in vivo (Fig. 4d, e). Moreover, it was unclear whether the identified CN activity was consistent with its expression. Western blot results showed that tutin did not affect CNA level in the hippocampus (Fig. 4f) or cortex (Fig. 4g), suggesting that tutin affects CN activity, not its expression. Collectively, the data confirmed that tutin activates CN in vitro and in vivo.

### CN inhibitor FK506 antagonizes epilepsy induced by tutin in vivo

**Fig. 5:**
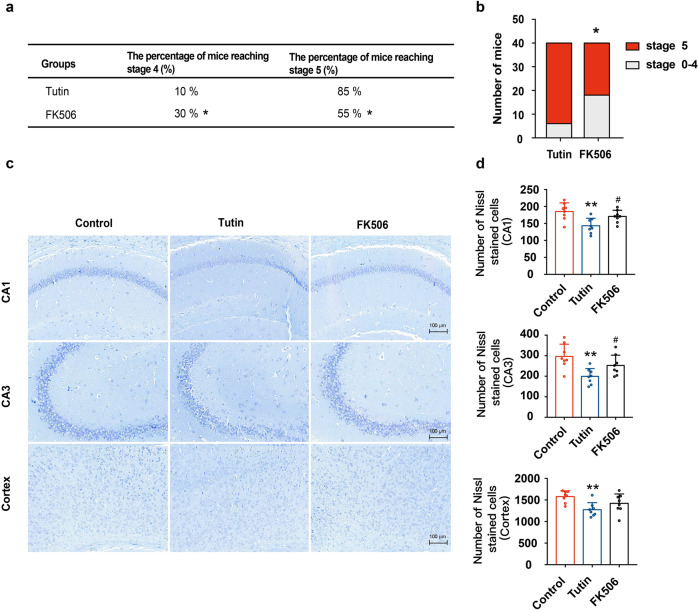
The CN inhibitor FK506 alleviates the intensity of seizures and reduces neuronal loss. **a** Percentage of tutin-induced seizures in mice with pre-treatment with FK506 (n = 40, *P < 0.05). **b** FK506 reduced the number of mice reached stage 5 epilepsy by tutin (*n* = 40, *P < 0.05). **c** Nissl staining of hippocampal CA1, CA3 and cortical neurons after tutin-induced seizures (scale bar: 100 μm). **d** Results of nissl staining were represented as mean ± SD with *n* = 8, **P < 0.01 vs. Control group, ^#^*P* < 0.05 vs. Tutin group

The CN enzyme-specific inhibitor FK506, was used to confirm that inhibiting CN activity reduces the stage of seizure induced by tutin. Seizure episodes induced by tutin showed typical increases in intensity. Compared with that in the tutin-challenged mice, pretreatment with FK506 obviously reduced the percentage of stage V seizures (Fig. 5a, b). Moreover, FK506 decreased the frequency of seizures and significantly reduced the duration of single seizure in EEG analysis (Supplementary Fig. S9). Since epilepsy can cause damage to neurons, particularly in the hippocampus,^5,39,40^ Nissl staining was performed in this study.^41^ The Nissl staining data showed that neurons were lost in both the hippocampus and cortex in the tutin-inuced mice, and FK506 significantly alleviated the injury of neurons in the hippocampal CA1 and CA3, while neurons in cortex were not significantly improved by FK506 (Fig. 5c, d). Other CN inhibitors had also been investigated, including Pimecrolimus and Cyclosporin. Consistent with FK506, Pimecrolimus significantly decreased the intensity of tutin-induced epilepsy. Cyclosporin showed similar effect on alleviating tutin-induced epilepsy in mice though without significance (Supplementary Tab. S4). Altogether, these data suggested that inhibition of CN activity may antagonize epilepsy induced by tutin, indicating that CN is a target of tutin during seizure induction.

### Knockdown of the CNA gene expression reduces tutin-induced epilepsy and neuronal damage in vivo

**Fig. 6:**
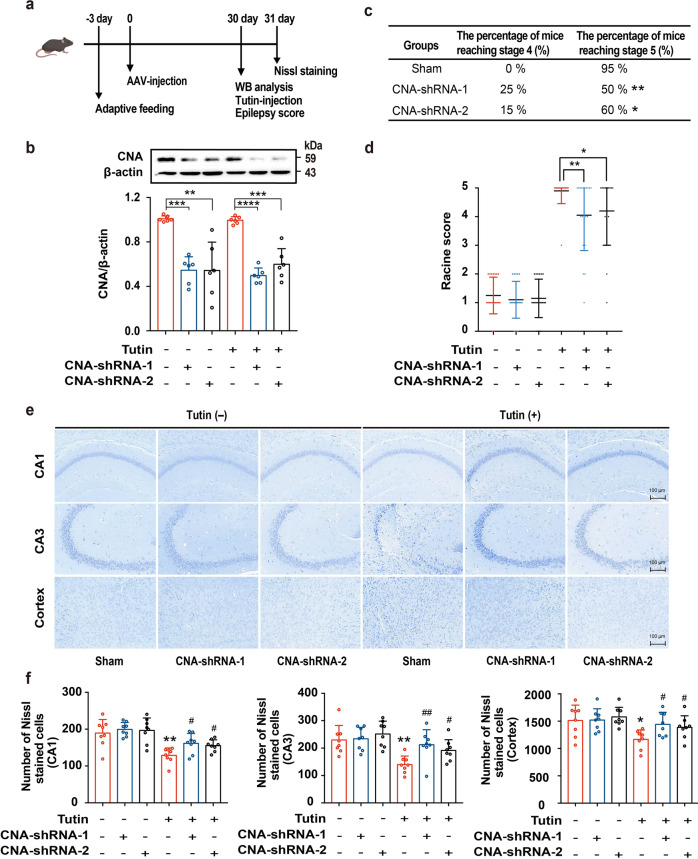
CNA knockdown reduces the intensity of seizures and neuronal loss. **a** Experimental flowchart was shown. **b** CNA expression in hippocampus by Western blot. Quantitative analysis of CNA in hippocampus of mice, and results were expressed as mean ± SD with *n* = 6, **P < 0.01, ***P < 0.001, ****P < 0.0001. **c** Behavioral episodes in mice with CNA knockdown induced by tutin (*n* = 20, *P < 0.05, **P < 0.01). **d** Distribution of maximum Racine score within 2 h after seizures, and results were represented as mean ± SD with *n* = 20 (*P < 0.05, **P < 0.01). **e** Nissl staining of hippocampal CA1, CA3 and cortical neurons after seizures (scale bar: 100 μm). **f** Statistical analysis of Nissl-stained cells of hippocampal CA1, CA3 and cortical neurons after seizures; Data are shown as mean ± SD with *n* = 8, *P < 0.05, **P < 0.01 vs.Tutin (-) -shRNA (-) group, ^#^*P* < 0.05, ^##^*P* < 0.01 vs. Tutin (+) -shRNA (-) group

To further verify that CN is a target of tutin in epilepsy, we knocked down *CNA* gene expression in the brains of mice. Our preliminary experiment indicated that CNA-short interfering RNA (siRNA)-1 and CNA-siRNA-3 could effectively decrease the level of *CNA* gene expression in N2a cells (Supplementary Fig. S11). Therefore, CNA-siRNA-(1/3) was applied to knock down CNA expression in vivo. Mice were microinjected with adeno‐associated virus (AAV)‐shRNA‐CNA into the left lateral ventricle, and sham control mice were injected with an empty AAV vector. After 30 days, the mice were injected with tutin, and seizure-like behaviors were observed (Fig. 6a). The expression of CNA in the hippocampus of the mice injected with adenovirus was significantly decreased (Fig. 6b). Behavior data showed that CNA-knockdown mice exhibited decreased intensity of the seizure attacks compared with sham mice (Fig. 6c). Moreover, the seizure score of the modified Racine scale was markedly decreased in the mice with CNA expression knocked down (Fig. 6d). The EEG results showed that CNA knockdown also inhibited the epileptic form discharges and significantly decreased the frequency of seizures (Supplementary Fig. S10). We then used Nissl staining to observe whether the reduction in CNA expression exerted a protective effect on neurons. The Nissl staining results showed that the neurons in the tutin-challenged mice were severely damaged in hippocampus (CA1, CA3) and cortex (Fig. 6e, f), while CNA knockdown significantly reduced the loss of neurons. These data further demonstrated that CN is a molecular target of tutin because knocking down CNA expression alleviated seizure severity and attenuated neuronal loss.

## Discussion

Targeted studies of natural products are critical for elucidating their mechanisms of action.^31,42,43^ As one of most importantly toxic molecules that cause epilepsy, tutin has been investigated to identify its neurotoxic targets through chemical proteomic approaches in this study. In our study, we demonstrated that tutin caused epilepsy in mice by activating CN with high neurotoxicity. Tutin bound CN by forming two hydrogen bonds in the active site to trigger CN activation in vitro and in vivo. It was confirmed that FK506 reduced the severity of epileptic seizures and degree of neuronal loss in mice treated with tutin. Furthermore, when CNA expression was knocked down in vivo, tutin-induced severe seizure rate was significantly reduced and nerve damage in mice with epilepsy was ameliorated. Therefore, we speculate that CN is an important target of tutin induction of seizures.

CN plays a central role in brain synaptic plasticity. Diazepam facilitates the inhibitory effects of GABA via targeting GABA receptors.^33,44^ Diazepam can significantly reverse tutin-induced seizures in mice, and it has been reported that CN downregulates GABA_A_ receptor function and that activated CN may interact with synaptic GABA_A_ receptors in the hippocampus CA1 inhibitory synapses.^34^ Injection of MK-801, a selective NMDA receptor antagonist, has been previously shown to block increases in CN activity, suggesting a role for calcium influx mediated by NMDA receptors in increased CN activity.^33^ In addition, martentoxin, a selective inhibitor for BK channel, exerted an antagonistic effect on epilepsy induced by tutin, suggesting that BK channel might be involved in tutin-induced epilepsy. In summary, tutin induces epileptic seizures in mice by activating CN, and NMDA receptors, GABA receptors and BK channels might be involved in related signaling pathways.

By employing various approaches, including TPP, CN inhibition and CNA knockdown, we verified that CN was, indeed, an important target of tutin during epileptic seizure induction. In neurons, CN plays an important role in epileptic seizures. During epileptiform activity, activation of CN has been reported to be related to *α*2 subunit containing GABA_A_ receptors. In the present experiments, we found that tutin activated CN in vitro and in vivo, and MST results suggested tutin bound to CN directly. The HDX-MS experiments, the molecular docking and MST analysis of two mutations indicated that tutin binds to the active site of the CN catalytic subunit. However, in contrast to the CN inhibitor FK506 binding site, the FK506-FKBP12 binary complex did not contact the phosphatase active site in CN.^45^ To better characterize the direct association between tutin and CN, further studies with other methods are needed.

Although our study verified that CN might be a possible potential target of tutin, we could not completely exclude the role of the other four proteins in the TPP-TR experiment. Literature search found that Aldh7a1 and Aldh3b1 are the members of Aldh, and Aldh7a1 has been linked to pyridoxine-dependent epilepsy.^46^ To explore the possible relationship between Aldh3b1 and tutin, the effects of pyridoxine were tested on tutin-treated mice. The results showed that pre-treatment with pyridoxine only had a certain auxiliary effect in epilepsy induced by tutin (Supplementary Fig. S12), suggesting that Aldh3b1 might not be a main target of tutin. The functions of Mvd, Lancl1, Aldh3b1 and Hsdl2 in epilepsy are still worthy to be explored in our future studies.

In summary, we explained, for the first time, the epileptogenic mechanism of the neurotoxic molecule tutin. We look forward to further exploring the activation mode of CN and the triggering mechanism of epilepsy to provide new strategies for the development of antiepileptic drugs in the future.

## Materials and Methods

### Reagents

Tutin (C_15_H_18_O_6_) was isolated from the roots of *Coriaria nepalensis* as described previously.^47^ Molecular weight of tutin was 294.11 and the purity of tutin was above 95%. The structure was identified by comparing its spectroscopic data (NMR and HR-ESI-MS).

### Primary cultured hippocampal neurons

Primary hippocampal cells were isolated from rats (Sprague Dawley, 24 h) (SiBeiFu Biotechnology, Beijing). The hippocampus was dissected out, and incubated with 0.05% trypsin (Gibco, USA) for 15 min at cell incubator, and then the suspension was filtrated through 70 μm filters. The cells were plated on 6-well plates (1 × 10^6^ cells) with Dulbecco’s modified Eagle’s medium (Gibco, USA), 10% fetal bovine serum (Gibco, USA), 100 U/ml penicillin and 100 μg/mL streptomycin (Invitrogen) overnight at cell incubator. After that, the cell medium was replaced by Neurobasal medium and cultured for 10 days. On day 10, monoclonal antibodies against NeuN (ABclonal, China) and MAP-2 (ABclonal, China) were used to assess the cell populations in the culture. Ten-day-old cultures composed of >95% neurons were used for this study (Supplementary Fig. S13).

### TPP

TPP experiments were performed as previous studies with minor modifications.^31^ Primary neurons were supplemented with tutin (5 μM) or PBS. Primary neurons were digested with trypsin, and suspended in PBS. Neurons were pelleted (600 × g, 5 min), and the supernatants were discarded. Neurons were resuspended in PBS (5 mL) and centrifuged (600 × g, 5 min), which were reconsistuted in 1 mL PBS. All the samples were separated into 10 fractions, and then heated at the following temperatures (37 °C-67 °C) for 3 min by Eppendorf Thermomixers. After that the samples were incubated at RT for 3 min, neurons were lysed with four freeze-thaw cycles (incubation at 35 °C for 30 min and snap-freezing by liquid nitrogen), and centrifuged (15,000 × g, 30 min) and then the supernatants were obtained for MS.

The concentration of 37 °C sample was measured and applied to normalize the volume for TMT labeling. After calculating the volume of each sample at 25 µg, 100 mM DTT (15.43 mg/mL) (8 M urea preparation) was added at 56 °C for 30 min. The protein samples were transferred to a pre-labeled 10 KDa ultrafiltration tube and centrifuged (15,000 × g, 20 min, 4 °C), and NH_4_HCO_3_ solution (150 µL, 50 mM) was added and centrifuged (15,000× *g*, 20 min, 4 °C). Iodoacetamide (20 mM dissolved in 50 mM NH_4_HCO_3_) and samples were incubated for 30 min, and then centrifugated for 20 min (15,000 g, 4 °C). NH_4_HCO_3_ solution (150 µL, 50 mM) was added and centrifuged (15,000 × g, 20 min, 20 °C), and then TEAB solution (150 µL, 100 mM) was added and centrifuged. TEAB solution was repeatedly added and centrifuged. Proteins were incubated with trypsin (1:50) for 12-14 h at 37 °C.

Peptides labeling was carried out by TMT reagents (Thermo Scientific), and anhydrous acetonitrile (41 µL) was added to dissolve TMT (0.8 mg). TMT solution (10 µL) was added to the samples (20 °C, 600 rpm, 1 h), and hydroxylamine (5 %, 8 µL) was added to quench the reaction, and then the labeled peptides were dried under vacuum centrifugation.

Peptide fractionation was carried out using an ACQUITY Arc Bio system (Waters) equipped with a Waters Bridge column (3.5 μm, 150 × 2.1 mm). The solvent A was 98% H_2_O 2% MeCN and 0.1% ammonia while the solvent B was 98% MeCN 2% H_2_O and ammonia. A 51 min gradient procedure was described as follows: 10 min 0% B, 0.1 min to 5% B, 1.9 min to 8% B, 11 min to 16% B, 21 min to 32% B, 1 min to 95% B, 1 min 95% B, 2 min to 15% B and 3 min 15% B, 400 μL/min, 214 nm. Fractions were dried, reconstituted in 1% FA in ddH_2_O, centrifuged (15,000 g, 4 °C, 30 min), and subjected to LC-MS/MS analysis. The experiment was performed in duplicates.

The peptides were tested by ultimate 3000 system coupled with an Orbitrap Fusion Lumos Mass spectrometer (Thermo Fisher Scientific, USA). The column of LC was an analytical column (50 μm, 15 cm) packed with 2 μm RSLC C18 (Mobile phase-A: water with 0.1% FA, B: 80% acetonitrile with 0.1% FA). The gradient was as follows: 5 min of 4% B, 4%-30% B for 65 min; 30%-80% B for 5 min; 80% B for 5 min; 80%-4% B for 5 min and 4% B for 5 min, 300 nL/min. The Orbitrap analyzer with 60,000 resolution (FWHM) was used to acquire the full scan MS spectra (m/z 350 to 1500). The elucidation of data was analyzed by Proteome Discoverer (2.3) workstation.

### CETSA

Neurons were heated at different temperatures (50-70 °C) for 3 min and lysed, which were separated by sodium dodecyl sulfate polyacrylamide gel electrophoresis (SDS-PAGE) and incubated with CNA antibody.

### CN activity assay in vitro

CN activity in vitro was determined according to the instructions of recombinant/purified CN activity kits (Abcam). Briefly, after calcineurin and calcineurin assay buffer were mixed, different concentrations of tutin are added for 5 min. The calcineurin substrate was added to start the reaction. This assay was then terminated by thegreen assay reagent (100 μL). The absorbance of OD_620_ was detected and the CN activity was calculated.

### Animals and treatments

C57/BL mice (male, 22–26 g) were supplied by SiBeiFu (Beijing) Biotechnology Co., Ltd. (China). Mice were raised at 24 °C with 12 h light/dark cycles, which is accessible to food and water freely. Mice were adapted for 7 days to before the experiments. All the experiments, especially epileptic seizure score experiments, antiepileptic drug therapy experiments, enzyme activity test, CN inhibitor assay, CNA knockdown experiments and Nissl staining tests were performed with the standard of double-blinded way. In antiepileptic drug therapy experiment, the mice were injected with tutin (2 mg/kg, i.p.) after oral administration of MK-801 (0.5 mg/kg), Retigabine (60 mg/kg), Diazepam (3 mg/kg), Carbamazepine (20 mg/kg). In the antiepileptic evaluation experiment of martentoxin, martentoxin (0.05 μg in 1 μL saline) was microinjected into the region of hippocampus, and then the mice were injected with tutin. In CN inhibitor assay, CN inhibitor FK506 (0.5 mg/kg, i.p.) was administrated to mice 1 h before tutin injection. The animal trails were carried out following the rules of the Institutional Animal Care and Use Committee of Peking Union Medical College. All procedures were carried out according to “three Rs” principle.

### Stereotaxic injection

Adeno‐associated virus (AAV)‐shRNA‐CNA were intracerebroventricularly (icv.) injected into the left lateral ventricle (Y: +0.5 mm, X: +1.0 mm Z: −2.5 mm) as previously reported. Anesthetized mice were fastened to a brain stereotaxic apparatus (RWD Life Science, China). For a single icv. injection of shRNA-CNA, 2 μL of adenovirus were injected and the microsyringe was kept for 5 min and slowly retracted. The wound was sutured, and then closed in layer with penicillin powder.

### Assessment of seizure

Epileptic activity of mice induced by tutin was scored for 2 h using a modified Racine scale as follows^48^: Stage 0: no reaction; Stage 1: facial clonus, including blinking, locomotor whiskers, rhythmic mastication, etc. Stage 2: including stage 1 and rhythmic nodding; Stage 3: fore clonus in addition to stage 2; Stage 4: including stage 3 and standing with hind legs; Stage 5: fall or jump, repeated convulsions or convulsions resulting in death on a stage 4 basis.

### EEG analysis

Male C57BL/6 mice were applied for EEG monitoring. Two electrodes were implanted, and one screw serving was inserted as the ground electrode into the skull through a drilled hole. The electrodes and screws were fixed with bone cement. Mice had a seven-day postoperative rest before the drug study. The mice were housed individually. A burst of high-amplitude EEG activity represents a seizure.

The baseline was measured for 0.5 h. The EEG signals in mice injected with vehicle or FK506 were measured for 1 h. Acute seizures were induced by intraperitoneal injection of tutin and the EEG signals were recorded for 150 min. The EEG signals in sham/knockdown mice injected with tutin were measured for 2 h.

### Microdialysis analysis

After fixation of mice, the microdialysis-guided cannula was inserted into the hippocampus (coordinate: A, −1.8; L, +1.5; H, −1.0 mm from bregma) and the cortex (coordinate: A, +1.5; L, +1.5; H, −1.0 mm from bregma) of mice. Microdialysis studies were conducted 7 days later. CMA 7 Metal Free probe (CMA, Sweden) with a 1-mm and 6-kDa-cutoff regenerated cellulose membrane was inserted gently through the CMA 7 guide cannula. The probe was equilibrated with artificial cerebrospinal fluid at a flow rate of 1 μL/min for 1 h prior to initiation of dialysis. After that, baseline samples were collected into vials for 30 min, then mice were injected with tutin and dialysates were collected every 30 min for 1 h. Microdialysis samples were dried under vacuum centrifugation, and re-dissolved with deionized water. After benzoylation reaction, the standard or samples were analyzed by LC-MS.

### CN activity assay

CN activity was measured following a modification of the procedures as previous studies.^49^ Briefly, the basal wells contained 25 mM MOPS (pH 7.0), 2 mM *p*-nitrophenol phosphate (*p*NPP), 1 mM DTT, 2 mM EGTA, and 2 mM EDTA. Other than that, the maximum wells contained 2 mM MnCl_2_. The final volumes of all wells were 100 μL. Brain region homogenate (200 μg/mL) was added to start the reaction at 37 °C for 0.5 h. The reaction was then terminated by placing on ice and OD was measured at 405 nm. The absorbance units were converted to the concentrations of *p*-nitrophenol (*p*NP) through comparing with a standard absorption curve of *p*NP concentration.

### Western blot

Brains were homogenized in lysis buffer and separated by 10% SDS-PAGE, and then transferred into PVDF membranes (Merck Millipore, USA). Membranes were incubated with skimmed milk (5%) for 1.5 h and then incubated with the an anti-rabbit antibody of CNA (ab109412, 1:1000, Abcam, USA) and β-actin overnight at 4 °C, which were incubated with the secondary antibody (1:20000, Santa Cruz Biotechnology, USA) for 2 h. All blots were developed with enhanced chemiluminescence regents (Merck Millipore, USA) and analyzed by Image J 1.53 software.

### Nissl staining

Mouse brains were fixed in the 4% paraformaldehyde, and then fixed with paraffin. After that, 3 mm brain slices were immersed in 1% cresyl violet (50 °C, 1 h) and dehydrated with different ethanol solution, and then brain sections were cleared with xylene. Nissl-staining cells of cortex, hippocampal regions were imaged by light microscope (NIKON E600, Japan) and analyzed by Image-Pro Plus.

### RNA interference

Neuro-2a cells were cultured in 12 well plates (10^4^/well). Negative control (NC) or CNA-siRNA were transfected by Lipofectamine^TM^ RNAiMAX transfection reagent for 48 h according to the instruction. CNA-siRNA sequence as follows: siRNA-1: 5′-GCCGTTCCATTTCCACCAAdTdT-3′; siRNA-2: 5′-GCGCTACTGTTGAGGCTATdTdT-3′; siRNA-3: 5′-GCAGTAATAGCAGCAATATdTdT-3′.

### CN expression and purification

DNA coding sequence of the human CNA/CNB (CNA: M1-N370, CNB: 16-170),^50^ was subcloned into a pET-15b (+) (Huada Gene BGI, Shenzhen) expression vector. The CN protein was expressed as described. Briefly, protein was expressed using *E. coli* strain Rosetta (DE3) (Tiangen Biotech (Beijing) Co., Ltd.) in 5 L Luria-Bertani medium induced with isopropyl-*β*-D-thiogalactoside (1 mM). Protein purification used a Ni-NTA Agarose column (Lot #163026181, Qiagen). Conditions for Ni-NTA affinity chromatography were 20 mM Tris-HCl, 10 mM imidazole, 150 mM NaCl, pH 7.5, as for the elution buffer, the concentration of imidazole was raised to 300 mM. The protein concentration and purity (>85%) were estimated using NanoDrop™ 2000 spectrophotometers (Thermo Fisher Scientific) and SDS-PAGE (Bio-Rad) respectively (Supplementary Fig. S14). CN was concentrated and stored at -80 °C.

### HDX-MS analysis

Before 2 h of HDX analysis, the compound tutin (200 μM) was added into the sample, with the control sample adding an equal volume of tutin buffer. For deuterium labeling, CN (4 μM) in the buffer (20 mM Tris-HCl, 1 mM CaCl_2_, 0.5 mM TCEP, and 150 mM NaCl, in H_2_O, pH 7.5) in the presence or absence of 200 μM tutin was diluted 10-fold by the labeling buffer containing 20 mM Tris-HCl, 1 mM CaCl_2_, 0.5 mM TCEP, and 150 mM NaCl, in 100% D_2_O at pD 7.4. After incubation for 30, 90 or 300 seconds at 25 °C, the same volume of ice-cold quench buffer containing 4 M guanidine hydrochloride, 500 mM TECP and 200 mM citric acid in water solution at pH 1.8, 100% H_2_O, was added to quench deuterium uptake. The sample was digested with pepsin (Promega) on ice for 5 min, and removed by centrifugation. An ACQUITY UPLC BEH C18 column (2.1 μm, 1.0 mm × 50 mm, Waters) equipped with an Ultimate 3000 UPLC system (Thermo Scientific) were used for the obtained peptides separation. A Q Exactive mass spectrometer was used for mass spectrometry analysis of the peptides. Mass spectrometry data were compared with Proteome Discoverer (Thermo Scientific) to match the corresponding peptide in CN. XCALIBUR (Thermo Scientific) was used to inspected peptide peaks. In order to estimate the max deuterium uptake of peptides, a repeated experiment was performed extending incubation in D_2_O for 24 h. HDExaminer (Sierra Analytics) was used for calculating deuterium uptake levels. Deut % for different peptides were calculated as follows.

$${{{\mathrm{Deut}}}}_i\% = \frac{{\# {{{\mathrm{D}}}}_i/(\# ( - {{{\mathrm{CO}}}} - {{{\mathrm{NH}}}} - )_i - \# {{{\mathrm{Pro}}}}_i - 1)}}{{{{{\mathrm{Max}}}}\,{{{\mathrm{D}}}}_i}} \times 100\%$$

# D_*i*_: deuterium numbers for peptide *i* at a certain hydrogen/deuterium exchange time; $\# ( - {{{\mathrm{CO}}}} - - {{{\mathrm{NH}}}} - )_i$: amide bond numbers of each peptide; # Pro_*i*_: the proline number for peptide*i*; Mxx D_*i*_: maximum deuterium uptake for peptide *i*.

### Molecular docking

The docking study was performed by using Schrodinger’s soft. The protein coordinates were retrieved from the Protein Data Bank (PDB code: 6NUU). The structures of tutin were generated and energy-minimized. Both the protein and the ligands were prepared by adding polar hydrogen atoms.

### MST assay

The proteins were labeled using the Monolith His-tag Labeling Kit RED-NHS 2nd Generation Kit. Tutin was diluted in a series of concentration. The labeled CN was diluted to working assay buffer (40 nM, 0.05% Tween-20). The mixture was incubated and then loaded into Monolith standard-treated capillaries. The thermophoresis was detected by A Monolith NT.115 instrument (Nano Temper Technologies) and *K*_D_ values was calculated by NT Analysis software (Nano Temper Technologies).

### Statistic analysis

Statistic analysis was performed using one-way ANOVA for multiple group comparison and unpaired Student’s t-test for two groups. The proportion of animals with stages 0-4 and stage 5 seizures was analyzed by χ^2^ test. *P* < 0.05 was regarded obviously significant.

## Supplementary information

Supplementary Information

Supplementary MS Data

## Data Availability

The data of this study are available as reasonable consultation with the corresponding authors.
