# Supplementary material for: The toxic natural product tutin causes epileptic seizures in mice by activating calcineurin
Source: Signal Transduct Target Ther. 2023 Mar 10;8:101. doi: 10.1038/s41392-023-01312-y (PMC9998865; doi:10.1038/s41392-023-01312-y)
Supplement: Supplementary file 1 — Supplementary Information [file 41392_2023_1312_MOESM1_ESM.doc]

Supplementary Materials for

**The toxic natural product tutin causes epileptic seizures in mice**

**by activating calcineurin**

Qing-Tong Han#, Wan-Qi Yang#, Caixia Zang#, Linchao Zhou#, Chong-Jing Zhang, Xiuqi Bao, Jie Cai, Fangfei Li, Qinyan Shi, Xiao-Liang Wang, Jing Qu^*^, Dan Zhang^*^, Shi-Shan Yu^*^

State Key Laboratory of Bioactive Substances and Functions of Natural Medicines, Institute of Materia Medica Peking Union Medical College and Chinese Academy of Medical Sciences, Beijing 100050, China

#These authors made equal contributions to this work.

^*^Corresponding authors. Tel.: +86-10-63165326, Fax: +86-10-63017757.
E-mail addresses: yushishan@imm.ac.cn (Shi-Shan Yu); danzhang@imm.ac.cn (Dan Zhang); qujing@imm.ac.cn (Jing Qu)

This WORD file includes:

Supplementary Materials and Methods

Figs. S1 to S14

Tabs. S1 to S4

**Supplementary Materials and Methods**

**General information**

Reagents and solvents were purchased from commercial suppliers. Anhydrous solvents were purchased from Innochem and stored under a nitrogen atmosphere with activated molecular sieves. ^1^H NMR and ^13^C NMR spectra were measured on Varian Mercury 500 (^1^H, 500 MHz; ^13^C, 125 MHz) spectrometers. Chemical shifts were given in ppm. Coupling constants were given in Hertz. High-resolution mass spectra for verification of synthesized small molecules were carried out on a Thermo Exactive Plus spectrometer. The ionization method was ESI and the mass analyzer type was orbitrap.

**Synthesis of the tutin probe**

C_19_H_21_NO_7_

Tutin (50 mg, 0.17 mmol) and 4-nitrophenyl chloroformate (34 mg, 0.17 mmol) were added to a round-bottomed flask, cover the rubber plug, and replace with nitrogen, 5 ml of DCM solution was added to fully dissolve it. Pyridine (34 mg, 0.43 mmol) was injected into a syringe, stirred at room temperature for 2 hours, the reaction was monitored and terminated by drying the solvent. The product was purified by preparing thin layers, dried and weighed. Then 4 ml of THF was added as the reaction solvent, DIEA 33 μL. Finally, propargylamine (9 mg, 0.17 mmol) was added dropwise, stirred at room temperature for about 1 hour. Then the solvent was removed under reduced pressure. The resulting residue was purified with chromatography (DCM/MeOH = 500/1-50/1, v:v) to give probe as colorless oil (31mg, 62%). The structure of probe was determined by HR-ESI-MS, ^1^H-NMR and ^13^C-NMR.

TLC (DCM/MeOH = 20/1): Rf = 0.49 [KMnO_4_]. ^1^H NMR (500 MHz, CD_3_COCD_3_) δ 7.08 (s, 1H, -NH-), 5.31 (s, 1H), 5.19 (s, 1H), 4.90 (s, 1H), 4.90 (s, 1H), 3.95 (s, 2H), 3.73 (s, 1H), 3.67 (s, 1H), 3.34 (s, 1H), 3.20 (s, 1H), 3.09 (s, 1H), 2.82 (s, 1H), 2.70 (s, 1H), 1.96 (s, 3H), 1.25 (s, 3H); ^13^C NMR (125 MHz, CD_3_COCD_3_) δ 174.2, 155.7, 141.5, 110.8, 80.4, 80.3, 77.7, 75.0, 71.5, 64.8, 60.1, 59.2, 51.8, 49.5, 49.3, 44.7, 30.1, 22.3, 19.8; HRMS (ESI) calcd for [M+H]^+^: 376.13974, found: 376.13809.

**Thermal protein profiling data analysis**

Prior to visualization of obtained output files, data were filtered using the following criteria:

(1) R^2^ > 0.8 for fitted curves for tutin and PBS treatment;

(2) Plateau of < 0.3 for PBS curves;

(3) Steepest slope of protein melting curves in paired set of tutin and PBS treated conditions < -0.06;

(4) Melting point difference for each protein between both PBS replicates < 1.5°C.

Following additional criteria were applied for hit identification:

(1) *P* value for the two replicate experiments is < 0.1;

(2) Melting point shifts for both paired replicates (tutin vs PBS) have the same direction;

(3) Melting point difference tutin vs PBS > PBS 1 vs PBS 2.

**Immunoﬂuorescent staining**

Primary hippocampal neurons were ﬁxed in 4% paraformaldehyde for 30 min, and then neurons were permeabilized with 0.1% Triton X-100. Neurons cells were incubated with rabbit anti-NeuN antibody at 4 ℃ overnight, and then incubated with FITC goat anti-rabbit IgG for 60 min at room temprature. The neurons were counterstained with 4’, 6-diamidino-2-phenylindole (DAPI, 1 μg/mL), and the images were captured by a Carl Zeiss microscope (Germany).


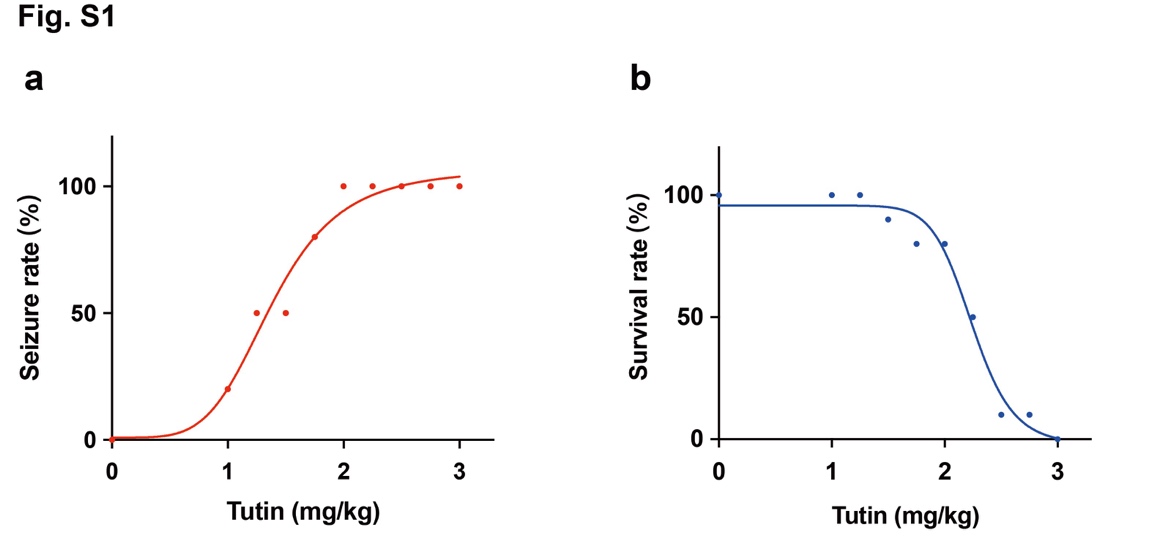
**Supplementary Figures**

**Fig. S1 The half convulsive dose (CD_50_) and half lethal dose (LD_50_) of Tutin were analyzed**. (a) Nonlinear dose-response curve in tutin-induced model of seizures was measured (ordinate shows seizure ratio, abscissa shows the dose expressed as mg/kg, n = 10). (b) Survival rate of mice after epileptic seizures caused by tutin was measured (ordinate shows survival ratio, abscissa shows the dose expressed as mg/kg, n = 10).

**
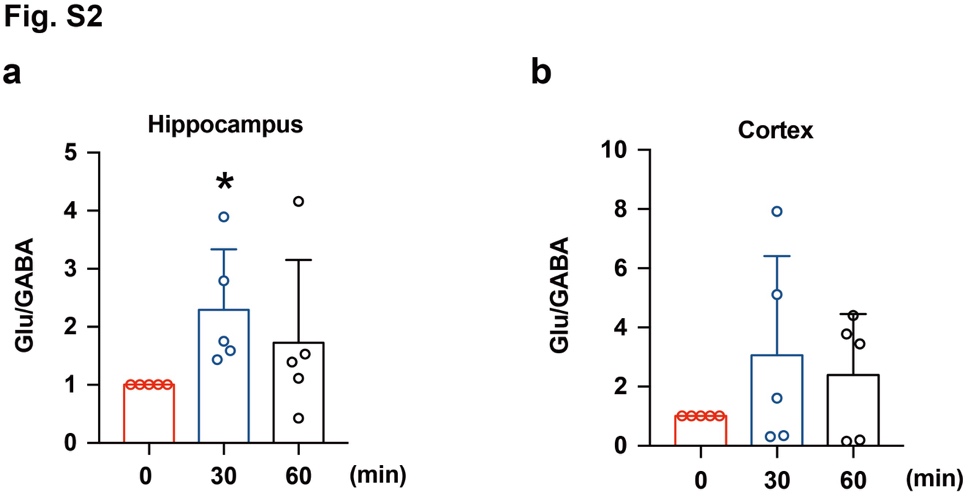
**

**Fig. S2** **Changes of Glu/GABA ratios before and after tutin-induced epilepsy in mice were shown**. The extracellular Glu and GABA of control and epileptic mice were derivatized and analyzed by LC-MS/MS. The ratios of Glu/GABA concentrations were measured in hippocampus (a) and cortex (b). Results were expressed as mean ± SD with n = 5 (**P* < 0.05, vs. 0 min).

**Fig. S3 The HR-ESI-MS spectra of tutin probe**

**Fig.S4 ^1^H-NMR-spectra of tutin probe in CD_3_COCD_3_**

**Fig.S5 ^13^C-NMR-spectra of tutin probe in CD_3_COCD_3_**

**Fig. S6 Thermal response curves were carried out using the TPP R package**. (a) Thermal response curves for Q62967 (Mvd) protein of Tutin-treated (solid lines) and PBS-treated (dotted lines) cells. (b) Thermal response curves for P63329 (CNA) protein of Tutin-treated (solid lines) and PBS-treated (dotted lines) cells.


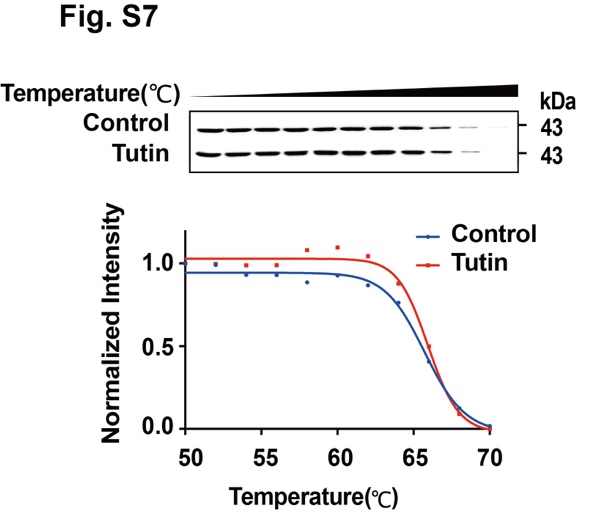
**Fig. S7** **Cellular thermal shift assay of β-actin control was measured by Western blot**. Tutin treatment (5 μM) did not affect the thermal stability of β-actin in cells as measured by the temperature-dependent cellular thermal shift assay (n = 3).


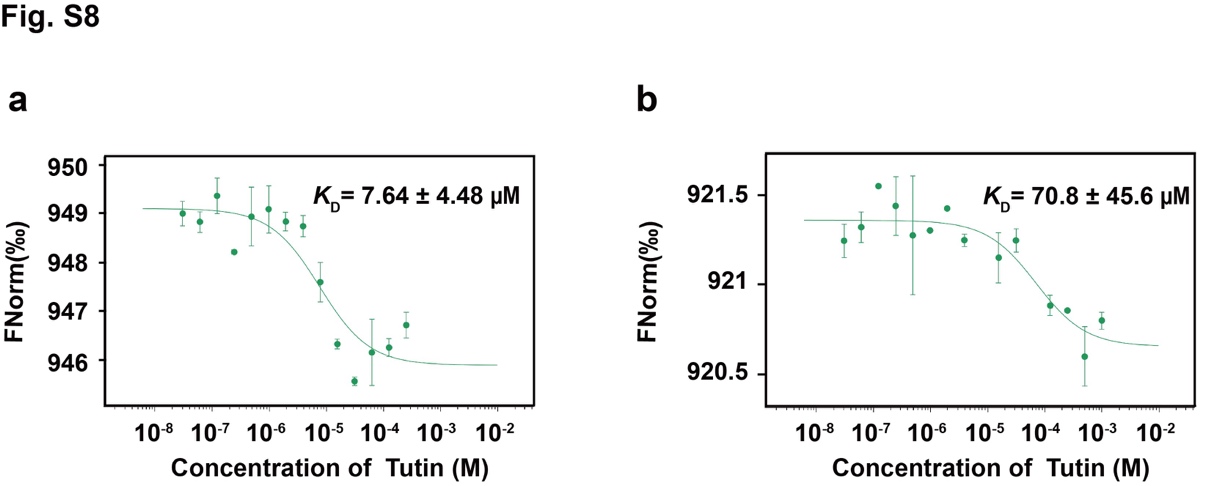


**Fig. S8 The interaction between tutin and CN-R254K or CN-A283V was measured by MST.** MST dose-response curves show the interaction between tutin and CN-R254K (a) or CN-A283V (b). Results were expressed as mean ± SD with n = 3.


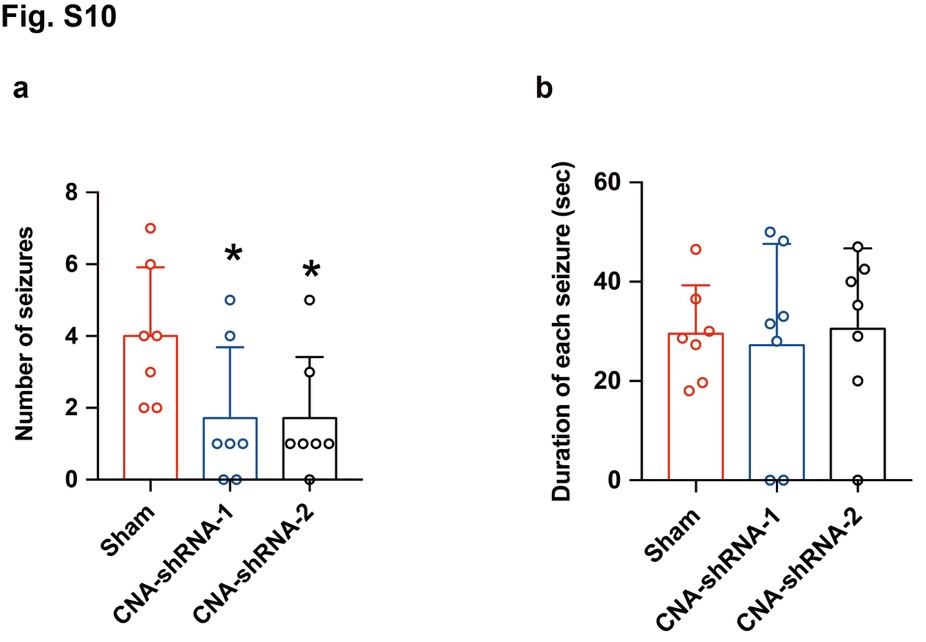

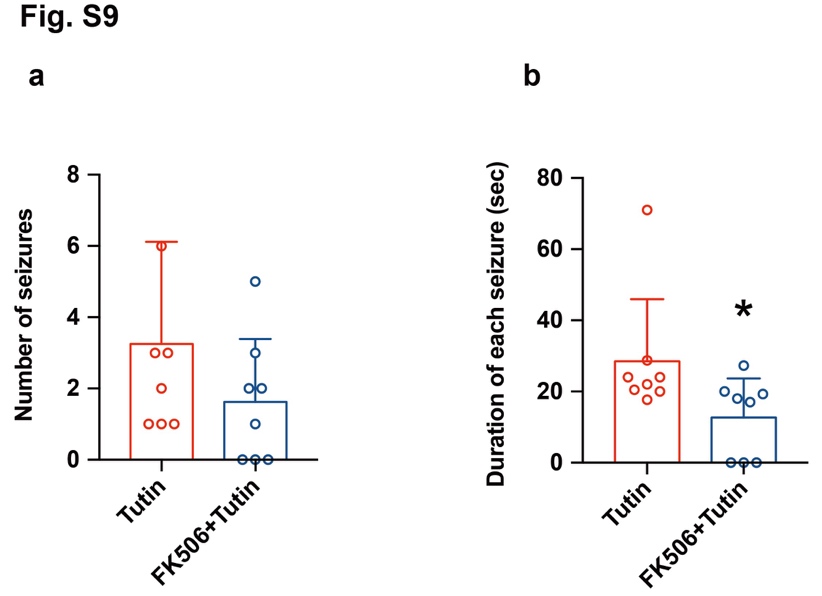
**Fig. S9 FK506 antagonized epilepsy induced by tutin in EEG assays**. Mice were pre-treated with FK506 (0.5 mg/kg, i.p.) 1 h before tutin injection. The EEG data were analyzed, and the total number of seizures (a) and mean duration of each seizure (b) are presented. Results were expressed as mean ± SD with n = 8 (* *P* < 0.05, vs. Tutin group).

**Fig. S10 CNA knockdown antagonized epilepsy induced by tutin in EEG assays**. Mice were microinjected with adeno‐associated virus (AAV)‐shRNA‐CNA or empty AAV vector into the left lateral ventricle. After 30 days, the mice were injected with tutin, and the EEG was recorded continuously. The EEG data were analyzed, and the total number of seizures (a) and mean duration of each seizure (b) are presented. Results were expressed as mean ± SD with n = 7 (**P* < 0.05, vs. Sham group).


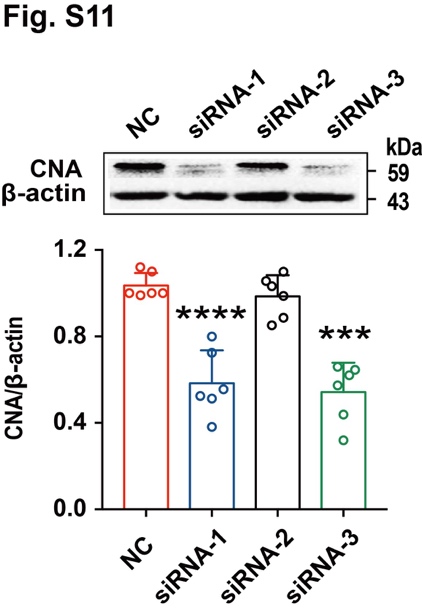
**Fig. S11 CNA was knocked down by short interfering RNA (siRNA) in N2a cells**. Preliminary experiment indicated that CNA-siRNA-1 and CNA-siRNA-3 could effectively decrease the level of CNA expression in N2a cells. Therefore, CNA-siRNA-1 and CNA-siRNA-3 were applied to knock down CNA expression *in vivo*. Immunoblots (top) and quantitation (bottom) were shown. Results were expressed as mean ± SD with n = 6 (****P* < 0.001, *****P* < 0.0001, vs. NC group).

**
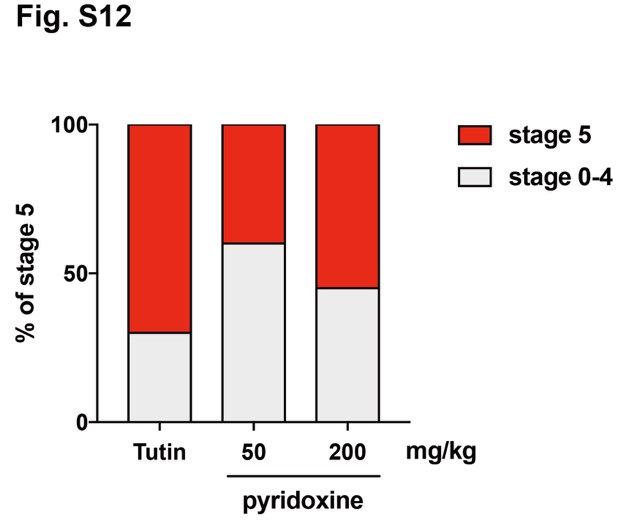
**

**Fig. S12 Effects of pyridoxine on seizures induced by tutin in mice were investigated.** Mice were pre-treated with Pyridoxine (Vitamin B6) 50 mg/kg and 200 mg/kg for 7 day，and then the mice treated with tutin were measured for behavioral seizures (n = 20).

**
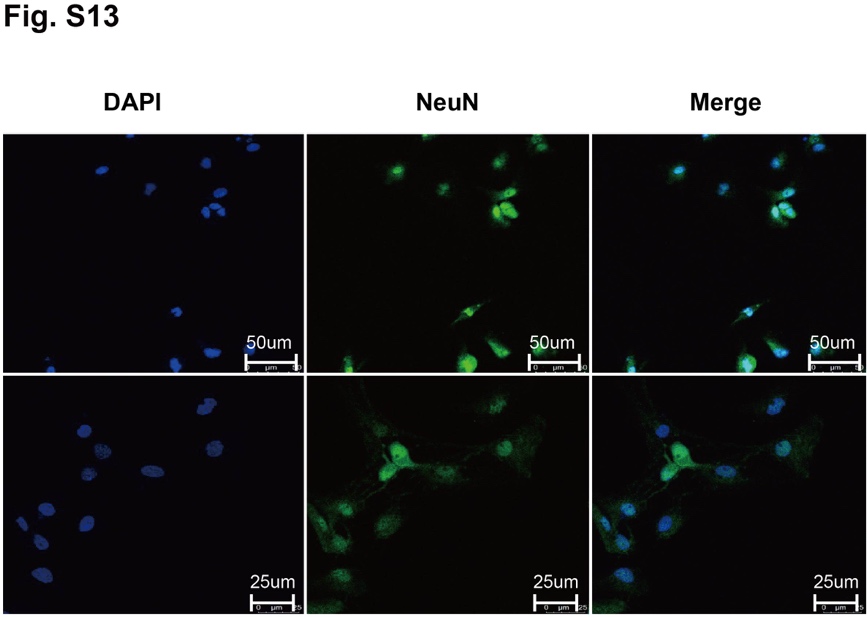
**

**Fig. S13 Representative immune-staining images of cultured primary hippocampal neurons**. The purity of primary cultured hippocampal neurons was more than 95% (n = 3). DAPI (Blue), NeuN (green).

**Fig. S14** **CN was assayed by coomassie brilliant blue staining**. Heterogeneous expression of CN (CNA 1-370, CNB 16-170) was assayed by SDS-PAGE.

**Supplementary Tables**

**Tab. S1 No behavioral changes induced by tutin probe**

| Tutin probe dose | Number of epileptic mice/Total mice number |
| --- | --- |
| saline | 0/8 |
| 1.0 mg/kg | 0/8 |
| 2.0 mg/kg | 0/8 |
| 3.0 mg/kg | 0/8 |
| 10.0 mg/kg | 0/8 |

**Tab. S2** Binding protein upon tutin treatment

| **Protein** | **Gene** | **diff_meltP _1** | **pVal_1** | **diff_meltP _2** | **pVal_2** |
| --- | --- | --- | --- | --- | --- |
| Diphosphomevalonate decarboxylase | Q62967 | 3.46902235 | 0.0027 | 2.74700458 | 0.0022 |
| Aldehyde dehydrogenase family 3 member B1 | Q5XI42 | 5.96321303 | 0.0005 | 1.88981159 | 0.55 |
| Serine/threonine-protein phosphatase 2B catalytic subunit alpha isoform | P63329 | 2.43893938 | 0.45 | 1.27685768 | 1 |
| Glutathione S-transferase LANCL1 | Q9QX69 | 2.06794351 | 0.19 | 2.19785503 | 0.12 |
| Hydroxysteroid dehydrogenase-like protein 2 | Q4V8F9 | 1.09208581 | 0.79 | 1.17325846 | 0.48 |

Recorded raw files were processed by Proteome Discoverer 2.4.1.15 (Thermo Scientific) software for protein identification and MS/MS spectra were searched against the Uniprot FASTA database for Rattus norvegicus (taxon identifier: 10116, downloaded on 1.7.2019). Downstream data analysis of corrected reporter intensities calculated by Proteome Discoverer in the protein groups table were further used for the determination of melting curves and Tm shifts, which was performed with R (version 4.0.2) and the TPP R package (version 3.16.5).

**Tab. S3 Altered peptides in HDX**

| **Peptides** | **Sequences** |
| --- | --- |
| 12-46 | STTDRVVKAVPFPPSHRLTAKEVFDNDGKPRVDIL |
| 62-72 | LRIITEGASIL |
| 81-86 | IDAPVT |
| 81-95 | IDAPVTVCGDIHGQF |
| 230-259 | ILWSDPLEDFGNEKTQEHFTHNTVRGCSYF |
| 232-242 | WSDPLEDFGNE |
| 243-258 | KTQEHFTHNTVRGCSY |

**Tab. S4 Antagonistic effect of CN inhibitors on tutin-induced epilepsy in mice**

| **Groups** | **The percentage of mice reaching stage 4 or above (%)** | **The percentage of mice reaching stage 3 or below (%)** |
| --- | --- | --- |
| Tutin | 83.33 | 16.67 |
| Pimecrolimus + Tutin | 33.33 * | 66.67 |
| Cyclosporin + Tutin | 66.67 | 33.33 |

Percentages of tutin-induced seizures in mice with pre-treatment of CN inhibitors were calculated (n = 12, **P* < 0.05 vs. Tutin group).
